# When Speech Signals Wilson Disease: A Case Report

**DOI:** 10.7759/cureus.116055

**Published:** 2026-09-10

**Authors:** Ali Abbas Zahid, Noor Ehsan, Muhammad Mansoor Ali, Hateem Ahmed, Usman Amjad

**Affiliations:** 1 Internal Medicine, Gulab Devi Teaching Hospital, Lahore, PAK; 2 Family Medicine, Gulab Devi Teaching Hospital, Lahore, PAK

**Keywords:** cirrhosis, dysarthria, gait ataxia, neurological type wilson disease, wilson disease

## Abstract

Wilson disease (WD) is a rare autosomal recessive disorder of copper metabolism that usually presents with a wide spectrum of seemingly unrelated symptoms, such as dysarthria, tremors, dystonia, features of advanced liver disease, and psychiatric manifestations. Atypical presentation may lead to delayed diagnosis. We present the case of a 25-year-old man who presented with slurred speech and abdominal distension. Workup revealed the presence of Kayser-Fleischer rings, reduced serum ceruloplasmin levels, elevated 24-hour urinary excretion of copper, and decompensated liver disease. Magnetic resonance imaging (MRI) of the brain was significant for altered signals in the basal ganglia, thalami, and periventricular white matter. He was diagnosed with WD and commenced on D-penicillamine and zinc therapy, with clinical improvement on follow-up. This case signifies that WD must be considered in any adult presenting with unexplained neurological symptoms. Early diagnosis is essential to prevent disease progression and complications.

## Introduction

Wilson disease (WD) is a rare autosomal recessive disorder of copper metabolism caused by pathogenic mutations in the ATP7B gene, leading to impaired biliary copper excretion and progressive accumulation of copper in the liver, brain, and other tissues [1]. If left untreated, this results in hepatic dysfunction, neuropsychiatric manifestations, and multisystem involvement [1]. The disease typically presents and manifests in the first three decades of life, although age and mode of presentation vary significantly [2].

The clinical spectrum ranges from isolated hepatic abnormalities to predominant neurological or psychiatric manifestations [3]. Neurologic involvement often includes dysarthria, dystonia, tremors, parkinsonism, and behavioral disturbances, which often cause a delayed diagnosis, especially in the presence of liver disease [2]. The absence of a single definitive diagnostic test further makes early detection challenging [4]. The usual diagnostic workup includes low serum ceruloplasmin, elevated 24-hour urinary copper excretion, liver function abnormalities, and supportive neuroimaging findings, interpreted using structured diagnostic criteria such as the Leipzig score [1,4,5].

Recent literature highlights the limitations and shortcomings of traditional biochemical tests and the role of evolving biomarkers and imaging modalities in improving diagnostic accuracy and precision [6]. Early recognition is important, as timely initiation of copper chelation therapy significantly improves prognosis and prevents irreversible organ damage [7]. In developing countries, including Pakistan, delayed diagnosis due to variable presentation and low awareness remains a concern, as reflected in recent local reports [8,9].

## Case presentation

A 25-year-old man presented to the outpatient department of Gulab Devi Teaching Hospital, Lahore, Pakistan, with a year-long history of gradually worsening speech difficulties. He complained of slurred speech and reduced speech volume. His voice had developed a prominent nasal quality, and his tone had become monotonous. This was associated with occasional drooling of saliva. There was no associated dysphagia, facial or limb weakness, tremors, or other neurological deficits. He also reported generalized, gradually progressive abdominal distention, associated with intermittent mild abdominal pain and generalized fatigue. There was no history of hematemesis, melena, pruritus, or prior blood transfusions. He had no significant past medical or surgical history, no history of substance abuse, and no family history of neurological or liver disease. The illness had significantly impacted his occupational and social life, causing him to eventually stop working.

According to functional assessment, he had effortful, slow, and slurred speech with a prominent nasal twang. He had no impairment in naming, repetition, reading, writing, or understanding commands. Comprehension remained intact. Neurological examination revealed intact motor power in all limbs, normal deep tendon reflexes, downgoing plantar responses, and absence of tremors or dystonia. He had a wide-based gait. The patient was icteric with mild pallor. The abdomen was protuberant with an everted umbilicus; the spleen was markedly enlarged, and shifting dullness was positive. The liver was not palpable, and hernial orifices were intact. Cardiovascular and respiratory examinations were unremarkable.

Laboratory investigations showed anemia, leukopenia, and thrombocytopenia. Liver function tests showed mildly elevated total bilirubin, alanine aminotransferase, and aspartate aminotransferase. Screening for hepatitis B and C viruses was negative. His coagulation profile revealed an elevated international normalized ratio (INR). Renal function tests and serum electrolytes were all within normal limits. His thyroid-stimulating hormone (TSH) was within normal limits. Both direct and indirect Coombs tests were negative. Serum ceruloplasmin was markedly reduced, and 24-hour urinary copper excretion was high (Table 1).

**Table 1:** Laboratory investigations

Investigation	Patient Value	Reference Range
Hemoglobin	9.9 g/dL	14-16 g/dL
Leucocytes	3.7 ×10⁹/L	4-11×10⁹/L
Platelets	56 ×10⁹/L	150-450×10⁹/L
Bilirubin	1.5 mg/dL	0.1-1 mg/dL
Alanine aminotransferase	64 U/L	7-56 U/L
Aspartate aminotransferase	78 U/L	8-48 U/L
International normalized ratio	1.91	0.8-1.1
Serum ceruloplasmin	5 mg/dL	20-60 mg/dL
24-hour urinary copper	328.3 μg/day	10-60 μg/day

Ultrasound of the abdomen revealed a mildly coarse liver echotexture, with no focal lesions or biliary dilatation, a dilated portal vein with a diameter of 15 mm (normal range: 9-13 mm), gross splenomegaly, 24 cm (normal range: up to 12 cm), and mild abdominopelvic ascites. Magnetic resonance imaging (MRI) of the brain with contrast demonstrated multiple areas of altered signal intensity in the bilateral periventricular white matter, centrum semiovale, and thalami, appearing isointense on T1-weighted images and hyperintense on T2-weighted and fluid-attenuated inversion recovery (FLAIR) sequences, without diffusion restriction, consistent with a metabolic disease process involving the basal ganglia and white matter. Kayser-Fleischer rings were identified on slit-lamp examination. An upper gastrointestinal endoscopy was performed, which showed grade 2 esophageal varices, for which band ligation was performed.

Following the diagnosis of Wilson disease, the patient was started on D-penicillamine 250 mg twice daily. He was later put on zinc 20 mg thrice daily. In addition, he was started on carvedilol 3.125 mg orally twice daily and a combination diuretic regimen of spironolactone 50 mg and furosemide 40 mg orally for management of portal hypertension and ascites. On follow-up visits during the past year, the patient has reported gradual improvement in speech and overall fatigue.

## Discussion

Wilson disease (WD) is a genetically inherited disorder of copper metabolism with highly variable clinical presentation, often leading to delayed diagnosis [10]. This case demonstrates a combined neurologic and subtle hepatic presentation in a young adult, highlighting the importance of early suspicion.

Isolated dysarthria is an uncommon presentation of neurologic WD [11]. Neurologic WD is typically characterized by extrapyramidal symptoms; isolated speech disturbance may be an early symptom, especially when white matter structures are involved [2,12]. Dysarthria in WD can occur due to involvement of multiple regions of the brain, such as the basal ganglia, cerebellum, the brainstem, and the corpus callosum [13-15]. Our patient’s presenting complaint was progressive dysarthria, which occurred in the absence of tremors, dystonia, or parkinsonism. His presentation highlights the importance of keeping WD in mind while investigating chronic progressive dysarthria, even in the absence of extrapyramidal symptoms [11]. 

The accompanying portal hypertension, splenomegaly, ascites, and pancytopenia all suggested advanced chronic liver disease [16]. Notably, transaminase elevation was mild, a pattern reported before in WD where progressive copper deposition leads to chronic architectural damage instead of acute hepatocellular inflammation [17]. Our patient’s transaminases were mildly raised, and yet he had advanced liver disease. This indicates the need to establish the presence of and stage of liver disease in patients with WD. It also goes on to show that WD should be considered in patients with even mild yet persistently deranged liver function tests [2]. 

Our patient’s markedly reduced serum ceruloplasmin, elevated 24-hour urinary copper excretion, and the presence of Kayser-Fleischer rings supported the diagnosis of WD. While ceruloplasmin alone lacks specificity [4], its combination with elevated urinary copper and the presence of Kayser-Fleischer rings meets established diagnostic criteria [5,18]. Neuroimaging findings of bilateral thalamic and basal ganglia hyperintensities also supported metabolic involvement [12]. Our patient’s brain MRI also showed altered periventricular white matter signals. White matter abnormalities on brain MRI are uncommon in WD [19]. White matter findings may be associated with impaired cognition in WD [15]. Interestingly, our patient had no cognitive impairment despite white matter involvement. More common MRI findings in WD include altered signals in the thalami and the basal ganglia [13]. 

Early initiation of D-penicillamine remains standard therapy and is associated with stabilization or improvement when started before irreversible injury occurs [2,7]. Research has shown that even severe neurological manifestations may be reversed and liver disease may become stabilized with treatment [20]. Our patient’s symptomatic improvement on follow-up is in line with reported outcomes.

## Conclusions

This case indicates that Wilson disease should be considered in young adults presenting with unexplained speech abnormalities, even in the absence of classical extrapyramidal movement disorders. Additionally, this case indicates that Wilson disease should be suspected in patients who have persistently deranged liver function tests, even when the derangement is mild. Early biochemical screening and prompt treatment remain essential to improving prognosis.
